# Understanding maintenance, repair, and replacement of prosthetic limbs using routinely-collected data: a retrospective study over three decades in Cambodia

**DOI:** 10.7189/jogh.15.04135

**Published:** 2025-04-25

**Authors:** Alex Dickinson, Lucy Gates, Cheryl Metcalf, Charlotte Spurway, Sisary Kheng, Thearith Heang, Bunthoeun Sam, Carson Harte, Sam Simpson, Peter Worsley, Chantel Ostler, Maggie Donovan-Hall, Amos Channon

**Affiliations:** 1 Faculty of Engineering & Physical Sciences, University of Southampton, UK; 2 Institute for Life Sciences, University of Southampton, UK; 3 Exceed Research Network, Lisburn, UK; 4 Faculty of Environmental and Life Sciences, University of Southampton, UK; 5 Faculty of Medicine, University of Southampton, UK; 6 Centre for Global Health and Policy (GHAP), University of Southampton, UK; 7 Exceed Worldwide, Phnom Penh, Cambodia; 8 Exceed Worldwide, Lisburn, UK; 9 Portsmouth Hospitals University NHS Trust, Portsmouth, UK

## Abstract

**Background:**

Prosthetic limbs deliver major quality of life and socioeconomic benefits for people with amputation, particularly in low-resource settings. The value of administrative data analysis is established for enabling sustainable health care improvement, but there has been limited research into the maintenance, repair, and replacement of prosthetic limbs. Survivorship data are sparse and highly variable, and rarely addresses differences between demographic groups.

**Methods:**

We investigated the distribution of time between device delivery, maintenance/repair, and replacement for a Cambodian cohort, considering the influence of a range of service delivery, user demographics, and health characteristics. We conducted Kaplan-Meier survival analysis and used a Cox model to compare repair and replacement likelihood between groups.

**Results:**

We explored 14 822 device deliveries to 6986 clients, with a median of three devices per person (interdecile range (IDR) = 1–9), and 22 878 repairs, with a median of one repair/device (IDR = 0–4). The median device survival before repair was 237 days (IDR = 38–854), and replacement was 727 days (IDR = 208–2154). Devices used by children and people in more active occupations were replaced earlier than those used by the population as a whole, upper-limb devices were replaced later than lower-limb devices, and devices were replaced earlier for volume change than for wear and tear. We observed several less intuitive trends. such as different preferences or capacities for device repair *vs*. replacement between clinics, and earlier device repair and replacement for women than men.

**Conclusions:**

Prosthetic limb maintenance, repair, and replacement are influenced both by the device’s durability and the user’s access to well-resourced physical rehabilitation services. A device that is worn-out and repaired or replaced early may indicate poor quality, or the opposite, *i.e.* that it fitted well and enabled great mobility. However, such analysis may enable us to identify groups who are less well-served by current devices or rehabilitation models and contribute to cost-effectiveness analysis of current services. Furthermore, the findings represent benchmark data against which engineers could measure new technologies, to ensure that innovation justifies its inherent risk by offering a genuine improvement which balances functionality, cost, and durability.

Negative emotional and socioeconomic impacts can arise for a person with amputation when their prosthetic limb ceases to fit, is damaged, requires maintenance, or fails [1–4]. Clinic or workshop visits during early rehabilitation are most associated with socket suspension due to discomfort, and volume and shape changes in the residual limb as it matures and stabilises following surgery. Replacements due to loss of socket fit may continue at lower frequency throughout the person’s life, and clinic visits are also required for repair and replacement of the other prosthetic components [5] whose certification requires a fatigue life over three million cycles [6] (**Figure 1**).

**Figure 1:**
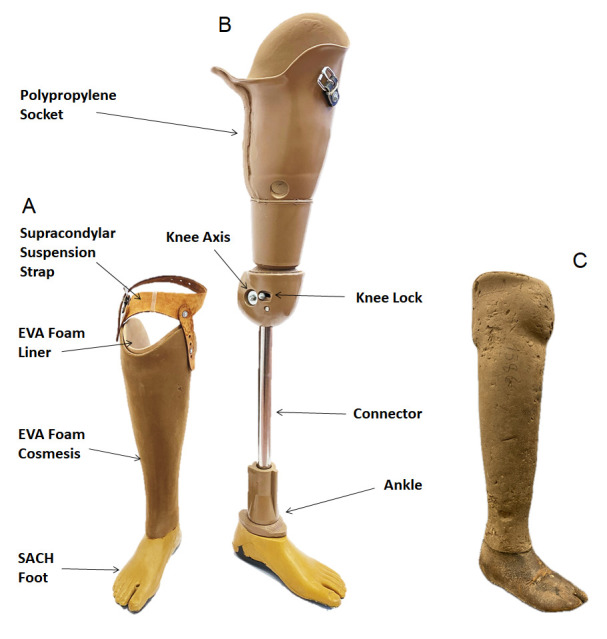
Example polypropylene technology prosthetic limbs produced by Exceed Worldwide Cambodia, according to ICRC Physical Rehabilitation Programme/Rehab’Impulse guidelines. Transtibial limb (**Panel A**) features a cosmetic foam shell which conceals the polypropylene socket, and transfemoral limb (**Panel B**) is shown without a cosmetic foam shell to reveal structural components. Transfemoral devices may also be used with a suspension belt, whose attachment buckle is shown. A transtibial device of PTB-SCSP design, in a worn condition (**Panel C**) typical of devices returned when clients are issued replacement devices. EVA – ethylene-vinyl acetate, PTB-SCSP – patellar tendon bearing supracondylar suprapatellar.

The socioeconomic benefits of prosthetic limb provision may be improved with evidence of the durability of the devices’ componentry and the socket’s capacity to accommodate fluctuations in residual limb volume and shape changes. However, this evidence is sparse and variable, and there have been relatively few activities for developing large-scale registries describing the care of people with limb absence [7]. This is in stark comparison to other health services that employ medical devices, such as orthopaedic implants, cardiovascular stents, and meshes for hernia repair, whose performance data are monitored, for example, in the Medical Devices Outcomes Registry (NHS England Digital) and several countries’ national joint replacement registries.

In the absence of detailed, comparable clinical data, administrative data may provide valuable insights [8]. Retrospective studies of prosthetic episodes in high-income settings [9–11] show lower limb devices lasting on average around two and a half years, with several prosthetist visits per year for socket adjustments or replacement, maintenance, and minor repairs, while upper limb devices usually last longer [12,13]. Data in low-resource settings generally show a longer time to device replacement, but these reports are sparse and less current [14–16], indicating a research gap that is important due to the potentially even greater socioeconomic impact arising from the poor function or failure of a person’s prosthesis [3], in these contexts.

On the whole, this research shows relatively consistent patterns of prosthesis maintenance, repair, and replacement across several settings, but indicates that the decision to repair or replace is not necessarily driven by device failure. There may be an influence of prescription and funding models, access to services, and (in some contexts) cultural factors which influence who is prioritised or encouraged to seek health care [14]. It is unknown whether some providers might make different decisions of whether to repair or replace devices depending upon their capacity or skills, and how readily their clients can access the prosthetics clinic [17]. Most of the above-cited studies present the mean number of visits per year or years of life per device, while those providing more detailed descriptive statistics report high variability within their cohorts, without investigating in depth the underlying reasons. At the time of writing, the literature contained limited consideration of device survivorship before replacement [18] and even less on survivorship before repair, with most data coming from low-resource settings being outdated and thus not being reflective of current technologies or practices in prosthetic care.

We aimed to analyse episodal statistics for a group of prosthetic limb users in Cambodia, to investigate the distribution of the time between device repair and replacement, and to consider variability by studying the influence of a range of the users’ characteristics. The research questions were defined as:

- What is the distribution in survivorship of prosthetic devices before repair or replacement?
- Are there inequalities between groups of users in how often an individual gets repairs to their prosthesis?
- Are there differences between groups of users in how often an individual receives a replacement prosthesis?
- Where trends are observed, are there potential health, social, political, environmental or technical explanations?

## METHODS

We undertook an observational retrospective analysis of routinely collected longitudinal data describing people accessing prosthetics services over multiple time points. The provision, maintenance/repair, and replacement of prosthetic devices were investigated. The data referred to clients visiting three clinics run by the non-governmental organisation Exceed Worldwide, in Phnom Penh, Kampong Som, and Kampong Chhnang, Cambodia, between 1992 and 2019 [19]. Approval was granted by national (230&311NECHR) and institutional ethics review boards (ERGO45577&51898) to analyse episodal statistics extracted from Exceed Worldwide’s digital clinical records. All records were collected in a standardised manner and stored in the ‘PMS-5′ database (International Committee of the Red Cross (ICRC), Geneva, Switzerland). Each line of data described a single clinical contact such as an assessment, a prosthetic device provision, maintenance/repair, or replacement. An individual client could have multiple contacts for the provision of prosthetic devices, and an individual device could have multiple maintenance or repair interventions (hereafter referred to simply as ‘repairs’).

We used two different groups from the administrative data (Table S1 in the **Online Supplementary Document**). First, we assessed all records from year 1992 onwards to understand the services provided over time. Second, we only included individuals who had used any service in the seven years (chosen based on the Exceed Worldwide Country Director’s recommendation (coauthor SK)) prior to 31 December 2019, classifying the group as ‘active’ users. Factors explored for inequalities were year of birth, gender, occupation or community role, type of prosthesis (as a proxy for limb absence level), year of prosthetic device receipt, reason/cause of limb absence/amputation, and, where relevant, the type of prosthesis repair and reason for replacement. Information about an individual on these dimensions was not routinely collected at every visit, so we used the first record for an individual that did record an answer, except for occupation/role, where we used the most recently recorded answer (as the occupation/role was likely to change). We grouped missing data into its own category within each factor, where appropriate, to ensure the sample size was not reduced.

As most data were skewed in their distribution, we presented medians and their interdecile ranges (IDRs), *i.e.* their 10th and 90th percentiles. We conducted a Kaplan-Meier survivorship analysis to describe the distribution of time until repair or replacement of devices. The median time between the replacement of prosthetic devices and the median time between repairs of the same device were calculated across different groups, and we plotted selected frequencies of repair per group time. We assessed differences between Kaplan-Meier estimates of survivorship for subgroups for significance at the 5% level by assessing overlap between the 95% confidence intervals for median estimates and using a log-rank test of equality between pairs of categories or a trend test where categories had a natural order (*e.g.* ages).

Kaplan-Meier analysis does not accommodate time-dependent variables or control for covariates. To assess the most important factors relating to replacement or repair of devices, we used the Prentice, Williams, and Peterson (PWP-GT) [20] recurrent event Cox proportional hazards model to estimate likelihood ratios (LRs) for repair and replacement separately. This approach is a conditional risk set model, accounting for correlations within the same individual or device for replacements or repairs, allowing the associations with other factors to be examined. Results indicate factors that are significantly related to an increased likelihood of a replacement or repair. Note that we use the term ‘likelihood ratio’ in the context of Cox models instead of ‘hazard ratio’ to denote that device repair or replacement is not always a negative event. Finally, we reviewed trends for potential health, demographic, and service delivery interpretations.

We performed all data analyses in Stata, version 17.0 (StataCorp LLC., College Station, Texas, USA).

## RESULTS

### Exploratory data analysis

After removing duplicate appointments on the same day for people with bilateral limb absence, the data set described 51 785 clinical contacts for 6986 individual clients, of whom 2894 were classified as active. Moreover, 14 822 full prosthetic devices were supplied, with an median of three (IDR = 1–9) devices per person (**Table 1**). Women were supplied with more devices than men, and there were no clear differences in the number of devices supplied according to a client’s year of birth. People with limb absence for congenital reasons had more prostheses than the rest (median of five (IDR = 2–14)), and people with upper limb amputations received fewer devices (median of one (IDR = 1–4) for transhumeral and two (IDR = 1–5) for transradial devices). Notably, people visiting the Kampong Som clinic received more devices (median of 6 (IDR = 1–13)) than clients visiting the other two clinics.

**Table 1:** Prosthetic devices delivered to all clients across characteristic groups and repairs per device, with count of deliveries or repairs in each group

	Deliveries		Repairs		
	**Median (IDR)**	**n**	**Median (IDR)**	**n**
**Year of birth**				
Pre-1940	2 (1–8)	218	1 (0–2)	205
1940–59	3 (1–8)	3556	1 (0–3)	4456
1960–69	3 (1–9)	3882	1 (0–4)	9416
1970–79	3 (1–8)	2253	1 (0–3)	3002
1980–89	3 (1–9)	1052	1 (0–4)	1758
1990–	4 (1–11)	861	1 (0–3)	997
**Cause of limb absence**				
Congenital	5 (2–14)	811	1 (0–3)	909
Traffic accident	3 (1–8)	943	1 (0–5)	1633
Weapon injury	3 (1–9)	11752	1 (0–3)	15490
Animal bite	5 (1–11)	213	0 (0–3)	251
Illness	2 (1–8)	478	1 (0–4)	687
Accident at work	3 (1–8)	323	1 (0–4)	466
Other	2 (1–6.5)	238	1 (0–3)	276
Missing	1 (1–5)	64	1 (0–4)	122
**Gender**				
Girls/women	4 (1–11)	2201	1 (0–3)	2805
Boys/men	3 (1–8)	12 621	1 (0–4)	17029
**Type of prosthesis**				
Partial foot	3 (1–8)	306	1 (0–3)	374
Transtibial (all)	3 (1–9)	11647	1 (0–3)	14996
Knee disarticulation	4 (1–8)	180	1 (0–3)	198
Transfemoral	3 (1–8)	2309	1 (0–4)	3836
Transradial	2 (1–5)	212	1 (0–3)	215
Transhumeral	1 (1–4)	45	1 (0–7)	115
Other	1 (1–5)	105	1 (0–2)	95
**Clinic**				
Phnom Penh	3 (1–7)	6510	1 (0–4)	11718
Kampong Chhnang	4 (1–9)	4039	1 (0–4)	6074
Kampong Som	6 (1–13)	4273	0 (0–1)	2042
**Overall**	3 (1–9)	14822	1 (0–4)	19834

The event records also contained 22 878 repairs (*i.e.* individual components repaired or replaced) on 19 834 different occasions (several repairs were to multiple different components on the same occasion). This represented a median of one repair visit per device, which was the same for almost all demographic and health variables, with IDRs from 0–3 or 0–4. Exceptions included many fewer repair visits for devices supplied by the Kampong Som clinic (median of 0 (IDR 0–1)) compared to the other two, and devices with more moving parts had a larger range in number of repairs than the rest (transfemoral with a knee joint (IDR = 0–4) and transhumeral with an elbow joint (IDR = 0–7)).

**Figure 2:**
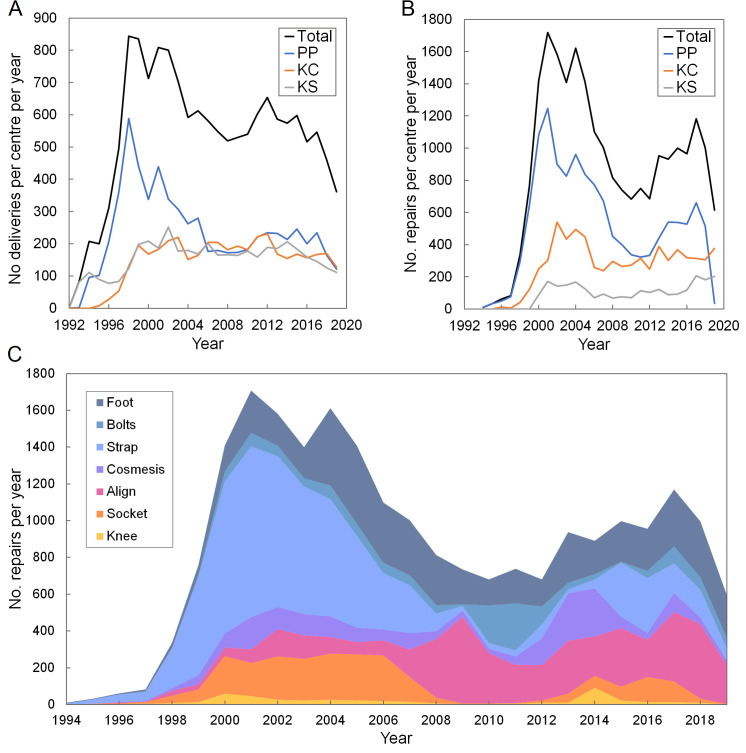
Time series graphs of the number of deliveries (**Panel A**) and repairs (**Panel B**) per year, showing the number for each of the three Exceed Worldwide centres and the total. Stacked chart (**Panel C**) of the number of different repair types provided over time for all three centres combined. KC – Kampong Chhnang, KS – Kampong Som, PP – Phnom Penh.

The time series data (**Figure 2**, Panel A) indicate a sharp rise in deliveries from 1992 to a peak of 710 devices in 1999, a stabilisation in the 2000s at around 500–600 devices per year, and a decline in 2018 and 2019. Similar trends are seen for the frequency of repairs over time (**Figure 2**, Panel B), with a ~ 2–3-year time lag. Since approximately 2006, the three clinics have delivered a similar number of devices, but a greater proportion of the repairs have been undertaken at the Phnom Penh clinic, and a lower proportion at the Kampong Som clinic. Overall, the Kampong Som clinic delivered 29% of the devices, including 34% of the replacements, but only 10% of the repairs, compared to the Phnom Penh clinic’s 44% of deliveries, 34% of replacements, and 59% of repairs.

### Types of repair and reasons for whole device replacement

From most to least prevalent, repairs included replacement straps, feet, adjustment of foot alignment, repairs and adjustments to sockets, replacement cosmeses and foot bolts, and replacements to knee, hand, or elbow components or their constituent parts (**Table 2**). The initial trend in repairs over time was dominated by strap repairs (**Figure 2**, Panel C).

**Table 2:** Different repair types, and replacement reasons, across the surveyed event records for all clients, presented as n (%)

	Phnom Penh	Kampong Chhnang	Kampong Som	Total
**Repair type**				
Replacement strap	5522 (40.9)	1409 (20.4)	227 (9.3)	7158 (31.3)
Replacement foot	2082 (15.4)	2384 (34.4)	523 (21.3)	4989 (21.8)
Alignment adjustment	1541 (11.4)	2062 (29.8)	726 (29.6)	4329 (18.9)
Socket repair/adjustment	1967 (14.6)	375 (5.4)	26 (1.1)	2368 (10.4)
Replacement cosmesis	776 (5.7)	491 (7.1)	698 (28.5)	1965 (8.6)
Replacement foot bolt	1151 (8.5)	112 (1.6)	166 (6.8)	1429 (6.2)
Replacement knee or component	370 (2.7)	43 (0.6)	39 (1.6)	452 (2.0)
Replacement hand or component	79 (0.6)	34 (0.5)	43 (1.8)	157 (0.7)
Replacement elbow or component	17 (0.1)	11 (0.2)	3 (0.1)	31 (0.1)
Total	13 505 (100)	6921 (100)	2452 (100)	22 878 (100)
**Replacement reason**				
Volume change	2094 (32.2)	2414 (59.8)	2859 (66.9)	7367 (49.7)
Wear and tear	1012 (15.5)	711 (17.6)	74 (1.7)	1797 (12.1)
Other/false info	1098 (16.8)	0 (0.0)	14 (0.3)	1112 (7.5)
Defined other*	49 (0.8)	29 (0.7)	52 (1.2)	130 (0.9)
Primary patient†	52 (0.8)	0 (0.0)	0 (0.0)	52 (0.4)
Missing	224 (3.4)	28 (0.7)	636 (14.9)	888 (6.0)
No replacement recorded	1981 (30.4)	857 (21.2)	638 (14.9)	3476 (23.5)
Total	6510 (100)	4039 (100)	4273 (100)	14 822 (100)

*Includes pain, stolen/lost, wrong alignment, and accident.

†This evidences a coding error, as this should not have been used as a reason for a replacement.

Of the 14 822 full devices that were delivered, 11 346 had a recorded replacement (**Table 2**). The most common reasons for device replacement were residual limb volume change and wear and tear. A small number of devices were replaced due to theft/loss, pain and poor alignment.

This time series data for repairs indicates several examples of how the service provider has used data collection in the past, responding to observations and improving device reliability by design. For example, the relatively short time before the replacement of leather suspension straps was identified by Exceed Worldwide around year 2000, and a decision was made to promote devices with alternative suspension thereafter (‘supracondylar’ PTB-SC and ‘supracondylar suprapatellar’ PTB-SCSP socket designs). The number of strap repairs then declined to a low level around 2009 once these devices had largely been replaced. A small burst of strap repairs was then made around 2015–17, which Exceed Worldwide attributed to a group of clients with highly demanding occupations requesting strap suspension for added security. Similar benefits of data collection arose in resolving purchased component quality issues. Exceed Worldwide reported anecdotally having observed an increased rate of foot bolt failure around 2010–11, which was attributed to a change in the material of the corresponding nut. As observed in the time series data, following the identification of a new supplier, bolt failure incidence returned to a low level. Similarly, perhaps an unexpected result was the short time to repair by knee component as this is a critical, moving, and highly-mechanically-loaded part of a transfemoral prosthesis. However, the numbers of knee failures were small and were attributed to the use of plastic in early knee extension block and flexion lock components. Around 2000, these were changed to metallic components and knee failures reduced.

### Device survivorship analysis

**Figure 3:**
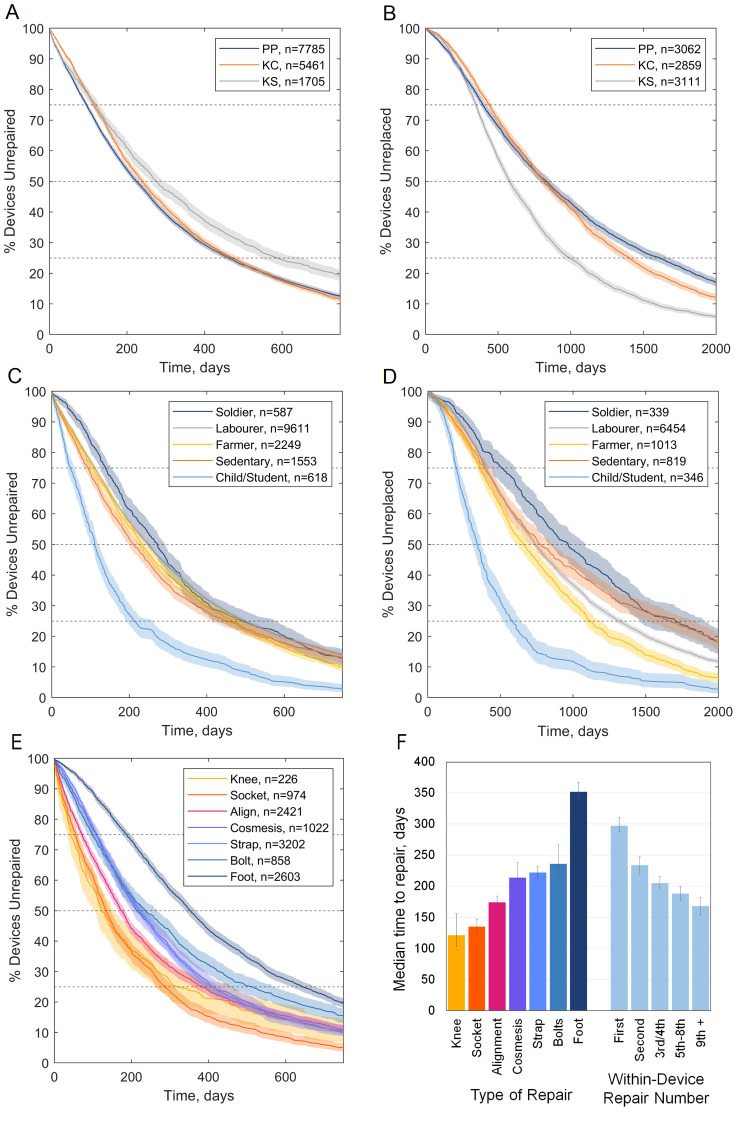
Kaplan-Meier estimates (**Panels A–E**) of time to repair (left) and replacement (right) of prosthetic devices, for active clients visiting the three centres run by Exceed Worldwide in Cambodia (**Panels A and B**), for client occupation or role (**Panels C and D**), and for noteworthy types of repair (**Panel E**). The latter is shown with a chart of median times to repair for those types of repair and numbers of repair within single devices (**Panel F**). On Kaplan-Meier charts, shaded zones indicate 95% CI and dashed lines enable the 25th percentile, median and 75th percentile times to repair to be compared between groups. KC – Kampong Chhnang, KS – Kampong Som, PP – Phnom Penh.

Considering active clients, 14 951 device repairs and 9032 device replacements were analysed. Overall, a quarter of devices had been repaired at 105 days, 50% had been repaired at 237 days and 75% had been repaired at 481 days (**Figure 3**, Panels A–E). For an end point of replacement, 75% of devices survived to 384 days, 50% of devices survived to 727 days and 25% of devices survived to 1299 days. Survivorship was illustrated using Kaplan-Meier graphs (**Figure 3**, Panels A–E), which show the estimated probability that devices were surviving unrepaired or unreplaced over time. On a Kaplan-Meier survivorship graph, a steeper line indicates that devices are being repaired or replaced sooner than a shallower line. Horizontal lines at the 75th percentile, median (50th percentile) and 25th percentile indicate the estimated time to repair or replacement of 25%, 50% and 75% of devices, respectively.

**Figure 4:**
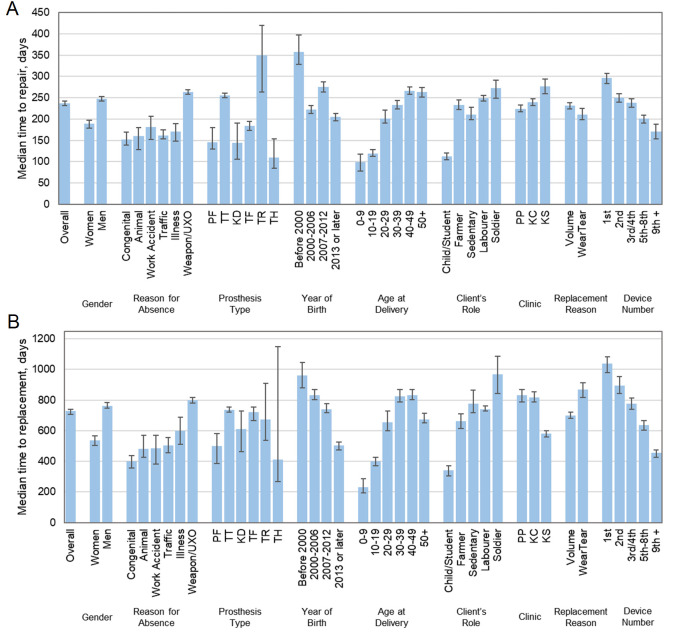
The median number of days between prosthetic device repairs (**Panel A**) and replacements (**Panel B**), for different population groups, Active clients only. Error bars represent 95% Confidence Interval. In repairs (**Panel A**), replacement reason refers to the prosthetic device’s ultimate reason for replacement and is not an indicator of how the device was repaired. KC – Kampong Chhnang, KD – knee disarticulation, KS – Kampong Som, PF – partial foot, PP – Phnom Penh, TF – transfemoral, TH – transhumeral, TR – transradial, TT – transtibial.

A summary of the time until 50% of devices were repaired and replaced is given in **Figure 4** for a variety of client demographic, health, and service delivery dimensions. Kaplan-Meier graphs illustrating noteworthy differences in time to repair and replacement for these subgroups are shown in **Figure 3**, Panels A–E. Where these dimensions showed no trends, or where survivorship trends were described adequately by the medians in **Figure 4**, Kaplan-Meier graphs are shown in Appendices (Figures S12–9 in the **Online Supplementary Document**). Finally, to control for confounding between variables, LRs for repair and replacement were calculated for the same demographic, health, and service delivery dimensions (**Table 3**; Figure S10 in the **Online Supplementary Document**). The LRs represent the probability that a particular device- or client group would receive a repair or replacement, relative to a reference case. For example, women had a device replacement LR of 1.07 (95% confidence interval = 1.01–1.14) compared to men, which was statistically significant (*P* = 0.027). The reference case for calculating the ratio within each group was chosen as the most frequent (*e.g.* gender, reason for limb absence) or earliest (*e.g.* decade of birth or number of device).

**Table 3:** The LR of prosthetic device repairs and replacements for different population groups*

	Replacement likelihood		Repair likelihood	
**Dimension**	**LR (95% CI)**	***P*-value†**	**LR (95% CI)**	***P*-value†**
**Gender**				
Men	1		1	
Women	1.08 (1.01–1.14)	0.021	1.07 (1.02–1.13)	0.008
**Reason for limb absence**				
Weapon/UXO	1		1	
congenital	1.12 (0.99–1.26)	0.071	0.92 (0.82–1.02)	0.112
Animal	1.29 (1.17–1.42)	<0.001	1.16 (1.08–1.25)	<0.001
Work accident	1.17 (1.02–1.35)	0.027	1.12 (0.96–1.31)	0.160
Traffic	1.27 (1.13–1.42)	<0.001	1.22 (1.11–1.34)	<0.001
Illness	1.36 (1.17–1.57)	<0.001	1.06 (0.95–1.18)	0.286
**Device type**				
Transtibial	1		1	
Partial foot	1.11 (0.94–1.31)	0.233	1.13 (0.98–1.31)	0.099
Knee disarticulation	1 (0.85–1.19)	0.978	1.05 (0.87–1.28)	0.560
Transfemoral	0.88 (0.82–0.94)	<0.001	1.06 (1.01–0.05)	0.011
Transradial	0.78 (0.62–0.97)	0.027	0.98 (0.84–1.16)	0.834
Transhumeral	0.76 (0.44–1.36)	0.371	1.18 (0.9–1.53)	0.240
**Year of delivery**				
Before 2000	1		1	
2000–06	1.18 (1.09–1.29)	<0.001	1.26 (1.15–1.36)	<0.001
2007–12	1.36 (1.25–1.52)	<0.001	1.08 (0.98–1.17)	0.138
2013 or later	1.82 (1.65–2.08)	<0.001	1.47 (1.32–1.61)	<0.001
**Age in years at delivery**				
0–19	1		1	
20–29	0.69 (0.6–0.79)	<0.001	0.62 (0.56–0.7)	<0.001
30–39	0.67 (0.59–0.77)	<0.001	0.64 (0.58–0.73)	<0.001
40–49	0.63 (0.55–0.73)	<0.001	0.59 (0.53–0.67)	<0.001
50–59	0.62 (0.54–0.73)	<0.001	0.57 (0.51–0.65)	<0.001
≥60	0.63 (0.54–0.75)	<0.001	0.57 (0.5–0.66)	<0.001
**Role**				
Labourer	1		1	
Child/student	1.2 (1.01–1.4)	0.035	1.19 (1.07–1.35)	0.002
Farmer	1 (0.9–1.04)	0.332	1.07 (1.01–1.12)	0.027
Sedentary worker	0.96 (0.95–1.1)	0.593	0.97 (0.9–1.03)	0.286
Soldier	1.08 (1.02–1.28)	0.022	1.06 (0.96–1.15)	0.257
**Centre**				
Phnom Penh	1		1	
Kampong Chhnang	1.23 (1.17–1.33)	<0.001	1 (0.95–1.04)	0.818
Kampong Som	1.43 (1.33–1.54)	<0.001	0.76 (0.71–0.81)	<0.001
**Replacement Reason**				
Volume changes	1		1	
Wear and tear	1.03 (0.96–1.09)	0.448	1.06 (1.01–1.12)	0.021
**Device no.**				
1st			1	
2nd			1.12 (1.06–1.19)	<0.001
3rd or 4th			1.3 (1.23–1.38)	<0.001
5th to 8th			1.48 (1.39–1.57)	<0.001
9th or later			1.7 (1.57–1.85)	<0.001

CI – confidence interval, LR – likelihood ratio, UXO – unexploded ordnance

*Within groups, the reference case is chosen as the most frequent (*e.g.* gender, reason for limb absence) or earliest (*e.g.* decade of birth or number of device). The ‘device number’ is not included as a variable in the repeated events replacement likelihood analysis due to the choice of the PWP-GT model, which uses the device number as the strata.

butSignificance set at a *P*-value of <0.05.

### Observed trends and interpretation: service provision dimensions

Considering service provision dimensions, we found a trend over time for device repairs and replacements to be provided earlier (Figure S2 in the **Online Supplementary Document**), and the likelihoods of repair and replacement have also increased (up to a LR of 1.46 and a LR of 1.86, respectively, for devices delivered since 2013 compared to pre-2000; *P <* 0.001 for both endpoints). If one assumes that devices are not becoming more unreliable, this may be explained by the prosthetics service becoming more established and better funded, equipped or staffed, and thus having greater capacity. Deviating from this overall trend, a longer time to device repair was observed from 2007–12 than in the periods on either side. This may be explained by the relocation of the largest of Exceed Worldwide’s three clinics at Phnom Penh between 2008 and 2010, including a period at a temporary site, which could have influenced clinic capacity and client awareness and prioritisation of providing new devices over repairs. This trend is also observed in time series data for the number of repairs and replacements conducted per clinic (**Figure 2**, Panels A and B). A slight drop in device provision is also observed in all three centres in 2018 and 2019, and a marked reduction in repairs at the Phnom Penh centre in 2019. This might be associated with a reduction in the available budget to support clients’ travel costs to access the service, which required Exceed to explore alternative service delivery models. In Phnom Penh, this included means testing for cost recovery.

The centres from which clients accessed their prosthetic care were also observed to influence the likelihood and time to device repair or replacement (**Figure 3**, Panels A and B). People served by the Kampong Som centre had a lower likelihood of having their devices repaired than the Phnom Penh and Kampong Chhnang centres (LR = 0.76; *P* < 0.001), and significantly later, but significantly higher likelihood of replacement (LR = 1.43; *P* < 0.001), significantly earlier.

**Figure 5:**
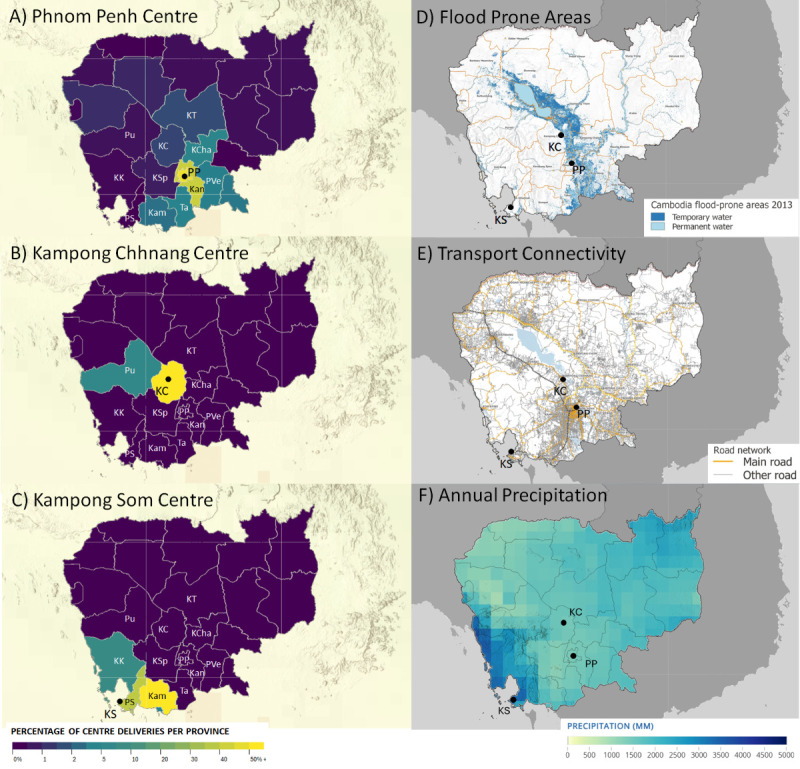
Geographic distributions by home provinces of clients to whom first devices were provided by the three centres run by Exceed Worldwide in Cambodia: Phnom Penh (**Panel A**), Kampong Chhnang (**Panel B**), Kampong Som (**Panel C**). Flood-prone areas of Cambodia (**Panel D**) and Road and railway networks in Cambodia (**Panel E**), both by Open Development Cambodia Learning Platform, accessed on 31 January 2025, licensed under CC BY-NC-SA 4.0. Heat map of average annual precipitation for 1995–2014 (**Panel F**), data sourced from the World Bank's Climate Change Knowledge Portal accessed on 31 January 2025, licensed under CC BY 4.0. KC – Kampong Chhnang, KS – Kampong Som, PP – Phnom Penh.

This may be explained by climate and transport factors (**Figure 5**, Panels A–F). The Kampong Som centre primarily delivered devices to clients from the Preah Sihanouk, Koh Kong, and Kampot provinces, which includes large rural areas with relatively low transport connectivity (**Figure 5**, Panel C). Preah Sihanouk and Koh Kong are also coastal and experience the county’s highest rainfall (**Figure 5**, Panel F), which will further expose the device to a more corrosive environment and interrupt transport. Provinces served by the Phnom Penh and Kampong Chhnang centres (Kampong Chhnang, Kampong Thom, Kampong Cham, Phnom Penh, Kandal, Prey Veng, and Takeo (**Figure 5**, Panels A and B)) lie in the Mekong Delta and are becoming more frequently affected by seasonal floods (**Figure 5**, Panel D) but have better road infrastructure (**Figure 5**, Panel E). Together, it appears that the sustained precipitation over a seven-month wet season in South West Cambodia, coupled with low transport connectivity, has a greater influence upon prescription practice or the wear experienced by devices before clients are able to bring them for intervention. As a result, a Kampong Som client’s prosthesis may be in a worse condition or beyond repair by the time they can visit the clinic.

Prosthetists may also preferentially replace devices instead of making more temporary repair interventions if their client is unable to return frequently or at short notice, due, for example, to travelling distance or the seasonal workload of subsistence farming [3,17], though analysis of client occupations or roles per clinic indicates relatively few of the Kampong Som clients were farmers (Figure S4 left in the **Online Supplementary Document**). Differences in the centres’ staff skill makeup and experience in repairing or replacing limbs may also have contributed, though all have International Society for Prosthetics & Orthotics-certified training.

### Observed trends and interpretation: demographic and health dimensions

Considering demographic and health dimensions, devices delivered to younger clients had a shorter time to repair and replacement, defined by age at device delivery and decade of birth (Figure S3 in the **Online Supplementary Document**). The difference was greatest for clients aged 0–9 years, perhaps due to their greater prevalence of limb absence or amputation for congenital reasons and their need for replacement devices due to their growth. While time to repair and replacement was positively associated with ‘decade of birth’, the time to repair and replacement was relatively uninfluenced by ‘age at device delivery’ for adults, *i.e.* individuals aged ≥20 years. Considering gender, women had significantly earlier repairs and replacements than men (Figure S5 in the **Online Supplementary Document**). This was partially related to their typically younger age than men, but when these confounding factors were removed, the repair and replacement likelihood remained higher for women (LR = 1.07; *P* = 0.009 and LR = 1.07; *P* = 0.027, respectively). Inspecting the clients’ occupations or roles, those described as ‘child’ or ‘student’ had their devices repaired and replaced significantly earlier than the rest (**Figure 3**, Panels C and D). After accounting for confounding factors, children/students had greater likelihood of repair and replacement than other community roles, reaching significance compared to the reference labourer group (LR = 1.20; *P* = 0.002 and LR = 1.19; *P* = 0.035 for repair and replacement, respectively). This corresponded with clients who were younger at device delivery; repairs and replacements were significantly less likely for all other age groups (LRs ranging from 0.57 to 0.65 for repair and from 0.63 to 0.69 for replacement; all *P* < 0.001).

Similarly, the typical older age of people with limb absence due to weapon or unexploded ordinance injury may explain why they had their devices repaired and replaced significantly later than the other reasons for limb absence, and people using devices due to congenital limb differences had their devices replaced significantly earlier (Figure S6 in the **Online Supplementary Document**). Controlling for age, these trends in likelihood of repair and replacement for these reasons for limb absence became less strong or disappeared (LR = 0.91; *P* = 0.115 and LR = 1.12; *P* = 0.067, respectively). This indicates that once a person with congenital limb absence reached adulthood, stopped growing and their residual limb stabilised, their devices performed similarly to the rest, or they required less frequent care.

Among adult clients, the device repair and replacement rate data were highly influenced by the predominant explosive weapon injury cause of amputation and relatively long time since amputation (Figure S6 in the **Online Supplementary Document**), characteristic of the Cambodian demographic [19,21]. However, when controlling for other variables, repair and replacement likelihoods were significantly higher for people with traffic accident injury (LR = 1.17; *P* < 0.001 and LR = 1.29; *P* < 0.001, respectively) and illness (LR = 1.22; *P* < 0.001 and LR = 1.26; *P* < 0.001, respectively; primarily diabetes and infection) than for weapon injury, and these are now the two most common reasons for amputations amongst Exceed Worldwide’s newly presenting clients in Cambodia [19].

Considering occupations of adult clients, devices used by farmers and labourers were replaced earlier than for sedentary people (**Figure 3**, Panel D). Farmers had a higher likelihood of receiving device repairs (LR = 1.06; *P* = 0.027). This might be associated with the mechanical forces and environment experienced in their physical work, which is supported by the observation that clients described as sedentary workers or ‘of few activities’ typically received device replacements later. Devices used by soldiers had longer time to device repair (*P* = 0.07) and replacement (*P* < 0.001), potentially because they are typically the older, more established device users in the study population, and represent the devices delivered earlier in the service’s time period (Figure S4 right in the **Online Supplementary Document**). Once correcting for confounding with related variables (number of device, age at delivery and delivery year), soldiers had a similarly increased likelihood of device repair to farmers, and a significantly higher likelihood of device replacement to other labourers (LR = 1.14; *P* = 0.022).

### Observed trends and interpretation: device dimensions

Transfemoral devices had significantly greater likelihood of repairs (LR = 1.06; *P* = 0.011), earlier, than transtibial devices (Figure S7, Panel A in the **Online Supplementary Document**), which can be explained by transfemoral devices’ greater technical complexity with more moving parts (*i.e.* a knee joint and lock) and use of accessories like a suspension strap or belt (**Figure 1**). There was no significant difference between the time to replacement for transtibial and transfemoral devices (Figure S7, Panel B in the **Online Supplementary Document**), however transfemoral devices did have a significantly lower likelihood of replacement (LR = 0.875; *P* < 0.000), possibly because their users are generally less active than people with below-knee prostheses [22]. Other device types showed greater variability in survivorship due to smaller sample sizes, but for upper limb devices, transradial limbs were typically repaired later, while more complex transhumeral limbs were repaired earlier. These upper limb devices were less likely to be replaced than lower limbs, reaching significance for the transradial level (LR = 0.77; *P* = 0.027). This may be associated with the much lower mechanical load placed upon upper- than lower limb devices, and the fact that clients with upper limb absence have more choice and opportunity to perform activities of daily living without using their prosthesis.

When a client had multiple devices, they were typically repaired and replaced progressively earlier (Figure S8 in the **Online Supplementary Document**). These trends may differ from expectations, where a client might need a large number of devices or repairs by socket adjustments during early prosthetic rehabilitation as their amputation heals and the residual limb volume and shape stabilise, and fewer devices as they become established users. This might be explained by people first accessing prosthetics services a longer time since amputation in low- than high-resourced settings [3,21], at which point their residual limb is more stable, if not yet biomechanically adapted to prosthesis use. With limb absence primarily due to trauma from weapons, unexploded ordnance and traffic accidents, clients in this population are more likely to survive for recovery into higher levels of device use than the people with vascular amputations who predominate in high income countries. Thus, they are more likely to return to work and physical activity, exposing their prostheses progressively to heavier wear. Clients who have been accessing prosthetic care for longer may also be more aware of what to ask for and more able to request changes due to discomfort. Further, clients in this data set may have previously been receiving care from another physical rehabilitation centre, and what is labelled as their first device is only their first received from an Exceed Worldwide centre [17]. This theory is supported by the repair likelihood data (**Table 3**; Figure S10 in the **Online Supplementary Document**), which showed a logical trend that multiple devices prescribed to the same person were more likely to be repaired, indicating progressively higher levels of use as the person becomes a more confident and established prosthesis user.

Finally, we considered individual device repair and replacement types. The survivorship analysis showed logically expected trends, that devices which would eventually be replaced due to wear and tear were significantly more likely to be repaired (LR = 1.06; *P* = 0.021), and sooner than those which would be replaced due to volume change and loss of device fit. Conversely, device replacement due to volume change occurred significantly sooner than replacement of devices which wore out (Figure S9 in the **Online Supplementary Document**). Survivorship analysis for different repair types reveals three phases of device maintenance (**Figure 3**, Panel F). Knee component adjustments and repairs, socket adjustments and device alignments were performed earliest (at a median of 121, 135, and 174 days, respectively), followed by replacement of cosmeses and suspension straps (at a median of 214 and 222 days, respectively), while device repairs by provision of a replacement foot component occurred latest (at a median of 236 days for the foot bolt and 352 days for the foot itself). The first group might comprise a large proportion of device adjustments in response to initial fitting, with over 15% occurring in the first month following provision, and a median time of around six months when Exceed Worldwide aims to provide their first scheduled follow-up for primary patients. Subsequently, cosmeses and straps are the first device components to be replaced, which is logical, as they employ soft materials, are exposed to the environment, and are subject to wear and tear. The prosthetic foot survives longest before repair despite its exposure to high levels of mechanical load and wear, because it is critical to the device’s function and therefore is designed to sustain these conditions. The locally made rubber foot has evolved for harsh environments, and design decisions to achieve longevity come at the expense of some biomechanical compromises [23]. The relatively early repair by replacing straps and cosmeses may also be due to opportunity, rather than an urgent response to device failure. Where a device had multiple repairs, these occurred progressively sooner (**Figure 10**, Panel B). Overall, although the ICRC Polypropylene Technologies/Rehab’Impulse knee components are functionally simple with their single axis lockable hinge, this study evidences their excellent durability with only 226 knee component repairs. Prior research indicates a rising incidence and prevalence of transfemoral amputations in Cambodia [19], so if engineers focus upon new knee prosthesis development for improved function, this data set sets them an important long-term durability target.

## DISCUSSION

### Summary of findings and comparison to published data

We analysed the likelihood and distribution of the time until prosthetic devices were repaired and replaced for a group of people with amputations or limb absence in Cambodia, considering the influence of a range of demographic, health, and service delivery factors. Overall, devices were replaced on average after 1.94 years, meaning clients had 0.52 device replacements per year. The average time to device repair was 0.65 years, or 1.53 repairs per year. These are similar to the previously reported data for high income setting services in the UK and the USA [9–12], and represent considerably more frequent intervention than the more historic low resource setting data [14,16,18], possibly due to Exceed Worldwide’s leverage of international resources and experience, and their established personnel, following their philosophy of investing in training local clinicians and technical staff.

Considering trends between groups, the observed higher repair and replacement rates for younger individuals was the same as found in two separate studies, both from a UK context, from 1999 [9] and 2001 [24]. The latter study was also able to identify that more repairs were performed for people with higher body weight, however, they saw no correlation between repair rate and gender, cause or level of amputation. Considering differences between devices, a 2008 UK study [10] found that transtibial devices were repaired and replaced more frequently than transfemoral devices, but that the latter had more socket replacements, enabled by device modularity aiding replacement of individual components (categorised in this study as repairs) instead of whole devices. They also observed insignificant trends for prioritising device replacement over repair in younger individuals with transtibial devices, and for more repairs and component changes in people with traumatic than dysvascular amputations. The present study agrees with prior findings, showing less frequent replacement of upper- than lower-limb devices, as the former experience lower mechanical loads, both in a 1982 low-resource setting study [14] and a 2015 analysis of veterans in the USA [12], though the difference in the average time before repair or replacement between upper- and lower limb devices was less marked in this data set.

Finally, at the component level, a smaller previous study found slightly higher survivorship of the foot, at 100% after six months and 80% after one year for the ‘HI’ vulcanised rubber solid ankle cushioned heel foot in Cambodia [23], which was listed as the most commonly used foot in our study (90% of repairs by foot replacement).

### The importance of further research into gender differences in prosthetic device outcomes

Gender presents an interesting and important dimension. It has been established that prosthetic service users are predominantly men in Cambodia and many other low-resource settings [19,25], but the implications are unclear. The observed higher likelihood of device repair and replacement for women might imply better access to physical rehabilitation services, greater health-seeking behaviour in women, or alternatively that they need more frequent care. This might be for health reasons or if the predominance of men using prosthetic devices has led to a situation where they have not been specifically designed to suit women’s requirements, and thus meet them less well. Recent studies indicate women are less likely both to have a major amputation and to be successfully fitted with a prosthesis [26], enter prosthetic rehabilitation later [27], and are under-represented in research [28]. Their greater likelihood of device repair and replacement may also link to differing societal roles and activity levels from men. The women in our data set were, on average, younger than the men at amputation and at device receipt, and more likely to be sedentary workers or students/children, and less likely to be labourers or farmers. However, their greater likelihood of device repair and replacement than men remained after adjusting for these confounding factors. Gender inequality is reported to be diminishing in cities in Cambodia [29], and no differences between women and men in employment opportunities for prosthetic limb users have been reported [3], though traditional societal norms persist in some rural communities, which may influence women’s opportunity to access prosthetics services. Our retrospective study cannot further distinguish between these possible reasons for gender disparities, and this important issue warrants further investigation.

### Limitations

Retrospective routine clinic data analysis is subject to inaccuracies arising from human input and interpretation of fields. There is some missing data, notably in the clients’ role or occupation, which may have been completed as their occupation at the time of the survey or at the point of amputation (Appendix S2 in the **Online Supplementary Document**). Further possible evidence of variations in how different staff and centres interpreted or used the form includes very low reports of wear and tear as a replacement reason from the Kampong Som clinic compared to the other two, and greater use of ‘missing’, ‘other’ or ‘false info’ returns at Phnom Penh and Kampong Som than Kampong Chhnang (**Table 2**). Differences in recording events between clinics, and potentially over time, may explain some of the reasons for their wide variation, and training is required to ensure similar approaches in recording data especially between centres and countries. That said, the study design should have minimised the risks of bias despite the long duration of data collection, as it employed routinely collected clinical data at the point of clinic visits. Selection bias was avoided as all clients were included in the centre’s electronic health record, though the survivorship analysis did consider active clients only, as described below. Recall bias is slightly more likely for a few dimensions such as the year of amputation, but otherwise all data were reported by the clinician or centre administrative staff during the visit.

Most devices were constructed by standardised techniques using comparable materials and componentry, which has demonstrated positive outcomes based on activity and quality of life in both qualitative [3] and quantitative [30] research. This avoids confounding between device technologies and social and health dimensions, but does prevent detailed comparison of devices and their suitability for different client groups. The study population does include a variety of feet, all of solid ankle cushioned heel design but obtained from different domestic manufacturers or import suppliers, for which a range of durability has been reported [23,31]. Our data set recorded the source of the replacement foot, but not that of the foot which was replaced. While a comparative analysis of the survivorship of the different foot types could have been conducted where a further repair to those replacement feet was recorded, this was precluded by very small sample sizes.

Moreover, our study does not give a definitive measure of the lifespan of prosthetic devices, because a long time to repair or replacement may not always indicate a positive outcome. This is similar to joint registries for orthopaedic implants [32], which have a definite end-point of revision surgery. This may be considered too high a risk, especially for patients who are elderly or have comorbidities. This associates with limitations of the Kaplan-Meier method, which assumes that censoring (individuals lost to follow-up) is non-informative, meaning that individuals who are censored have the same likelihood of experiencing the repair or replacement event as those who remain in the analysis. In this study, there are several reasons devices may have stopped being used without being repaired or replaced. Some clients may have stopped using their prostheses, died, or taken their nationally-standardised polypropylene technology devices for repair or replacement at centres other than the three included in the study [17]. To avoid clients lost to follow-up from skewing the results, only devices with a record of replacement were considered in the survivorship part of the study for active clients. To further mitigate these limitations, we supplemented the survivorship analysis with the PWP-GT recurrent event Cox model for repair and replacement likelihood. The PWP-GT model is particularly suitable for this analysis due to the recurrent nature of the data, where prosthesis users enter and exit the data set at different times and can have multiple experiences of replacement or repair on the same device. Unlike the standard Cox model, which assumes independence of ‘failure’ times, the PWP-GT model was used to accommodate the dependence between successive repairs and replacement of the same device. While the proportional hazards assumption can be violated in long-term data sets, such as this over-20-year study, the PWP-GT model's structure helps mitigate this issue compared to a standard Cox model by accounting for within-subject correlation [33]. Additionally, stratifying by event order helps address potential proportional hazards assumption violations, and sensitivity analyses confirmed the robustness of results as removing variables did not significantly alter outcomes.

### Interpreting the time to device repair and replacement: an optimal time window

Care must be taken over interpreting early repair or replacement of a heavily worn device as always representing a negative outcome. Heavy wear may indicate a device is comfortable and functional, and hence is heavily used, which anecdotally prosthetists report as a success. The wear may reflect the individual’s activity level, occupation and environment; a device worn by a more sedentary office worker will not be the same as that of an agricultural worker. A poorly fitted, uncomfortable device will not exhibit the same degree of wear. Additionally, device condition and repair or replacement are influenced both by the need for and the opportunity to access a prosthetics clinic. A relatively prompt repair or replacement can indicate that an individual has good access to prosthetics care, without necessarily indicating a shortcoming in device design or fabrication quality. Our study can only provide limited evidence to analyse these technical factors, or the barriers preventing physical rehabilitation centre access. The study also captures only repairs conducted in a formal rehabilitation service setting, whereas the importance of more informal and community-based repair is becoming appreciated, especially in remote and lower resourced settings [4].

Similarly, a very long time to repair or replace might be considered an undesirable outcome if it results from the person not using their device. There may, therefore, be an optimal range of time for replacement; this may differ between groups, for example, with earlier replacement required by growing children, or people with vascular amputations who experience higher fluctuations in residual limb size and greater soft tissue vulnerability to poor socket fit. The intra-national variation identified in this one-country, three-centre study also indicates that there may be different optima between settings. Analysing these data together with conversations with service providers may enable distinction between early maintenance and repairs or replacements which were opportunistic, and those which were associated with premature device failure or loss of fit, so that the cause can be addressed. This might help identify how long a device ideally lasts, subject to typical use and access to services for repair or replacement. Thus, we might define when a device has been repaired or replaced ‘too soon’, especially for clients of particular occupations, implying some premature failure, or indeed ‘too late’, implying some insufficient access to care.

## CONCLUSIONS

The potential value and risks of the use of ‘big data’ for health improvements in low- and middle-income countries are now well established [34]. However, the scientific literature reporting of prosthetic limb survivorship before repair or replacement remains sparse and shows high variability. The present study offers a highly detailed analysis of a relatively large and heterogeneous population of individuals within a single country, all receiving devices from the same service-providing non-governmental organisations, and comparable devices constructed from ICRC-initiated polypropylene technologies with solid ankle cushioned heel feet.

The high variability of survivorship measures is to be expected with great variety in prosthesis user characteristics and, unlike an orthopaedic implant, a device whose continued use is the person’s choice. Unlike some other medical devices, it would be overly simplistic to draw the conclusion that a longer prosthetic limb lifespan is inherently positive and a shorter lifespan is negative. Such analysis will enable comparison with other user groups and settings and may enable us to identify people who are less well-served by current devices or rehabilitation models, as well as modify physical rehabilitation services to address these inequalities towards universal health coverage. Device survivorship data may also contribute to cost-effectiveness analysis of current services across many more settings, which is of particular importance for reporting back to government and charitable funders [35] and justifying sustained funding to provide continuity of care.

Finally, this study provides benchmark data against which engineers could measure new technologies. All new health care technologies present a risk, no matter how well-intentioned. The potential impacts of this risk are most severe for vulnerable people in LMIC communities [36], and it is our responsibility to ensure that prosthetics technology innovation offers a genuine improvement above established devices.

## Additional material


Online Supplementary Document
