# Supplementary material for: Understanding maintenance, repair, and replacement of prosthetic limbs using routinely-collected data: a retrospective study over three decades in Cambodia
Source: J Glob Health. 2025 Apr 25;15:04135. doi: 10.7189/jogh.15.04135 (PMC12023805; doi:10.7189/jogh.15.04135)
Supplement: Online Supplementary Document [file jogh-15-04135-s001.pdf]

Supplement to:
Dickinson A, Gates L, Metcalf C, Spurway C, Kheng S, Heang T, Sam B, Harte C, Simpson S, Worsley P, Ostler C, Donovan-Hall M, Channon A. Understanding maintenance, repair, and replacement of prosthetic limbs using routinely-collected data: a retrospective study over three decades in Cambodia. J Glob Health. 2025;15:04135. https://doi.org/10.7189/jogh.15.04135

Appendix 1: Additional Data

Table S1: Raw characteristics of people accessing Exceed Worldwide services for prosthetic assessment, prosthetic device provision, repair, and replacement. Percentages include missing data. There are small differences between these two groups, with active clients more likely to be women, seen in Kampong Chhnang clinic, and younger.

		**All**	***N=6986***	***Active***	***N=2894***
		**(%)**	***Count***	**(%)**	***Count***
**Gender**	Women	12.6	*879*	15.0	*434*
	Men	87.4	*6107*	85.0	*2460*
**Clinic**	Phnom Penh	47.9	*3346*	41.5	*1200*
	Kampong Chhnang	21.9	*1528*	29.5	*855*
	Kampong Som	30.2	*2112*	29.0	*839*
**Year of Birth (Age at end 2019, years)**	Before 1940 (>79)	3.1	*215*	1.5	*42*
	1940-1959 (60-79)	26.6	*1861*	25.2	*729*
	1960-1969 (50-59)	43.4	*3031*	40.4	*1170*
	1970-1979 (40-49)	15.4	*1075*	13.3	*385*
	1980-1989 (30-39)	6.4	*446*	9.7	*280*
	1990 and later (≤29)	5.1	*358*	*10.0*	*288*
**Age at first consultation (years)**	0-19	1.4	97	2.4	*69*
	20-29	5.0	352	6.7	*195*
	30-39	20.1	1405	17.8	*516*
	40-49	35.6	2488	30.2	*873*
	50-59	21.4	1492	21.7	*629*
	60+	16.5	1152	21.2	*612*
**Reason for limb absence / Cause of Amputation**	Congenital	3.4	*235*	4.6	*133*
	Road Traffic Accident	6.7	*466*	11.1	*320*
	Weapon Injury	75.3	*5262*	64.6	*1868*
	Animal Bite	1.0	*71*	1.4	*40*
	Illness*	5.8	*408*	8.1	*233*
	Accident at Work	2.6	*181*	3.3	*94*
	Other	2.7	*190*	4.4	*190*
	Missing	2.5	*173*	2.7	*78*
**Role or Profession (earliest reported)**	Labourer/ Mobile Labourer	53.8	*3759*	*58.4*	*1691*
	Farmer	4.3	*302*	*9.3*	*268*
	Sedentary Worker/Person with few act	10.2	*713*	*9.6*	*278*
	Soldier	9.5	*661*	*7.1*	*206*
	Child/Student	2.2	*154*	*4.6*	*132*
	Other	0.8	*53*	*1.7*	*49*
	Unknown/Missing	19.2	*1344*	*9.3*	*268*
**Type of prosthesis supplied, as a proxy for level of limb absence or amputation**	Partial Foot	2.5	*72*	1.5	*42*
	Transtibial**	61.4	*4287*	63.9	*1850*
	Knee Disarticulation	1.0	*72*	1.5	*42*
	Transfemoral	20.5	*1430*	18.5	*535*
	Transradial	4.8	*337*	3.6	*104*
	Transhumeral	1.5	*105*	1.6	*45*
	Other	1.7	*116*	1.7	*50*
	No Prosthesis Supplied	6.7	*465*	6.8	*196*

* Illness for all clients includes diabetes (20.8%), disease (41.9%), gangrene (3.9%), infection (31.6%). Illness for active clients includes diabetes (30.9%), disease (45.9%), gangrene (1.3%), infection (20.2%).

** Transtibial includes PTB (patella tendon bearing), PTB SC (supracondylar) and PTBSCSP (supracondylar, suprapatellar).

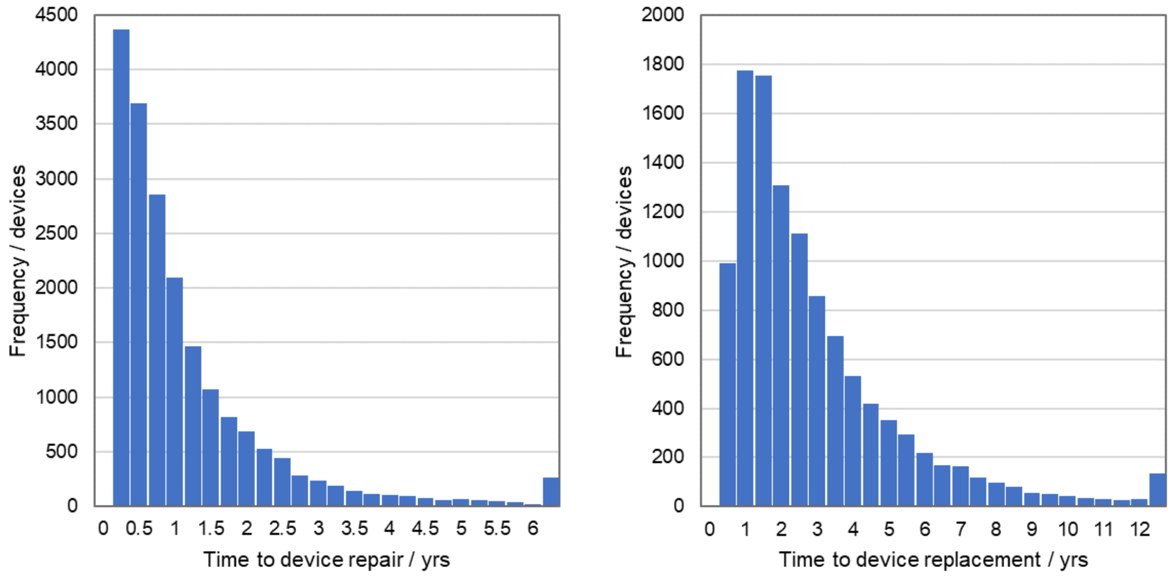

Figure S1: Histograms of time to repair (left) and replacement (right) of prosthetic devices, for all clients

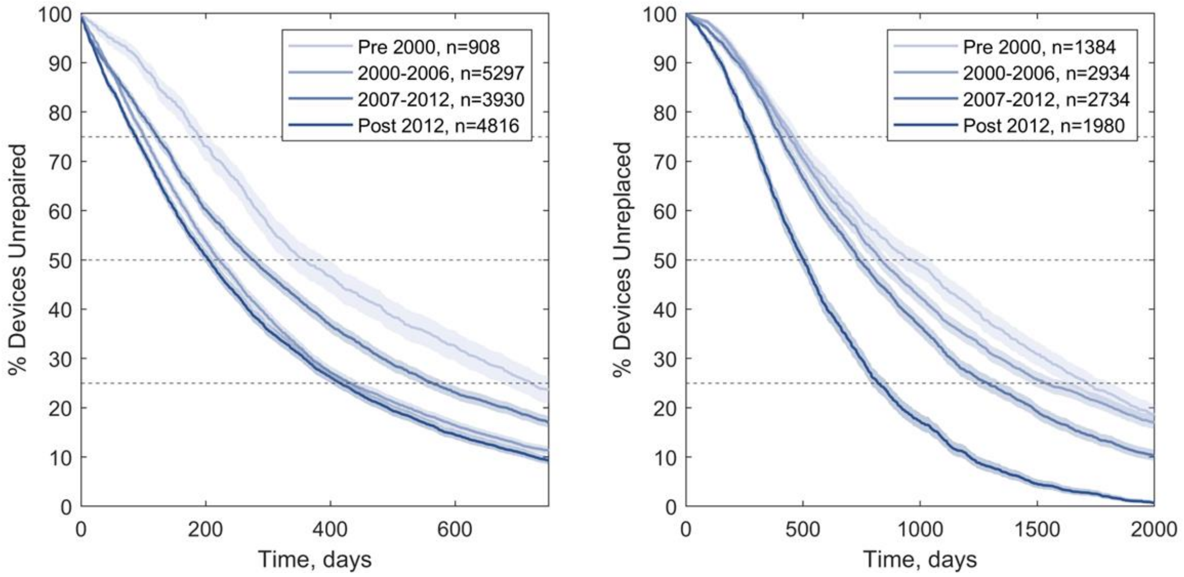

Figure S2: Kaplan-Meier estimates of time to repair (left) and replacement (right) of prosthetic devices, for devices categorised by their year of delivery (top). Shaded zones indicate 95% C.I. and dashed lines enable the 25th percentile, median and 75th percentile times to repair or replacement to be compared between groups.

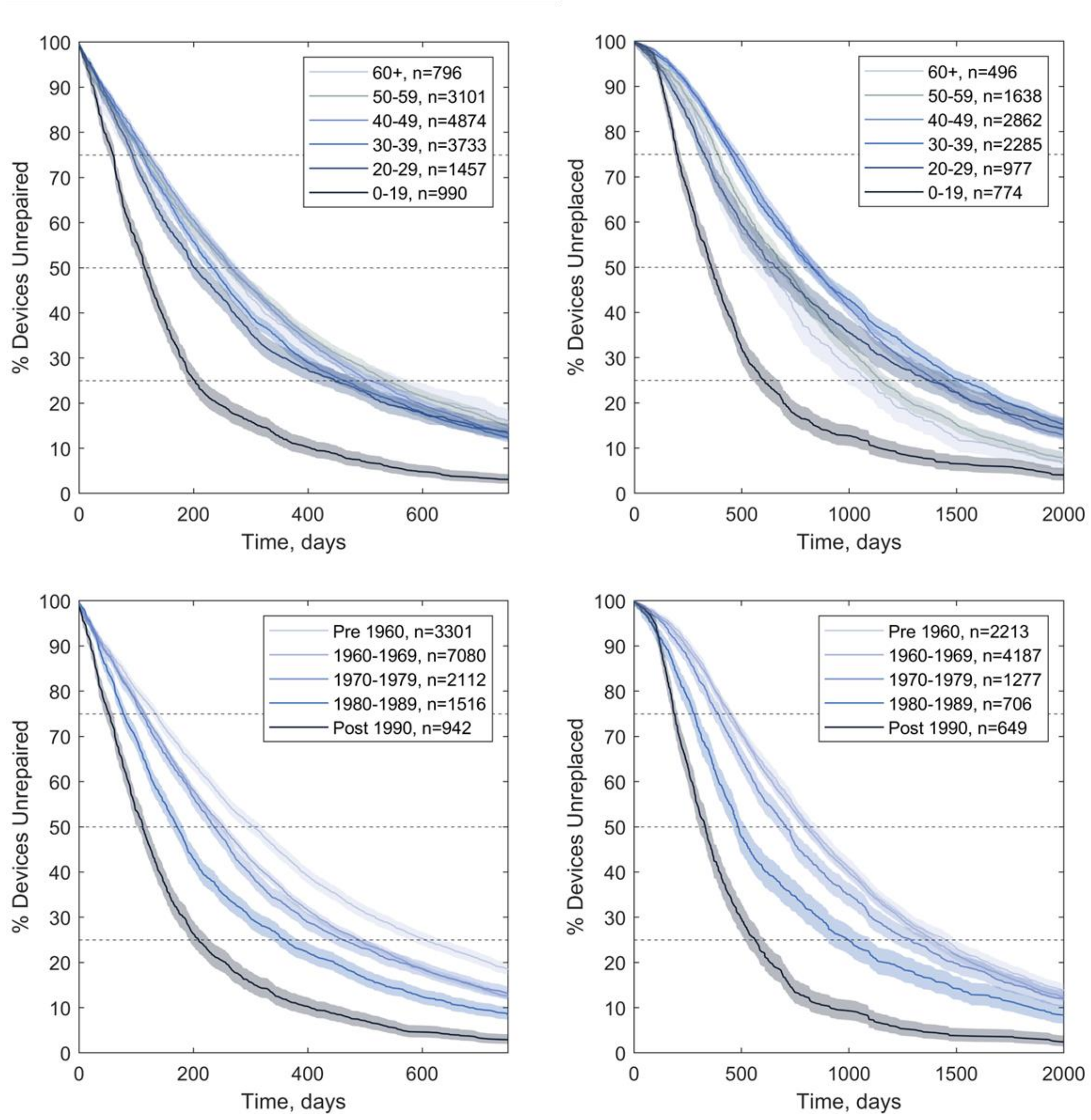

Figure S3: Kaplan-Meier estimates of time to repair (left) and replacement (right) of prosthetic devices, for active clients grouped by their age at device receipt (top) and their birth decades (bottom). Shaded zones indicate 95% C.I. and dashed lines enable the 25th percentile, median and 75th percentile times to repair or replacement to be compared between groups.

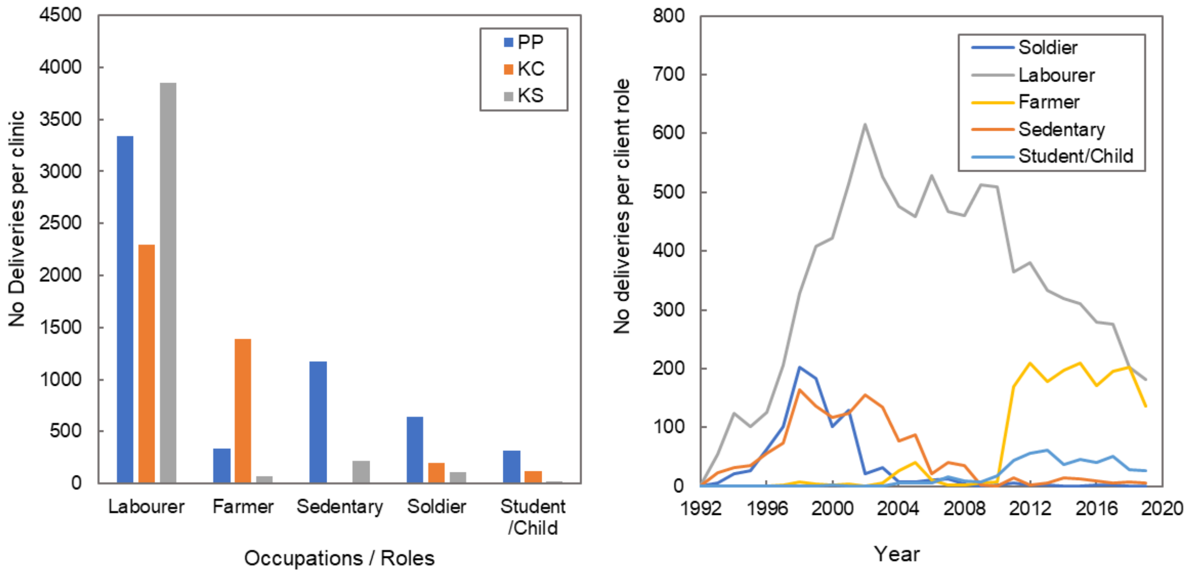

Figure S4: Distribution of client occupations or roles across the three clinics (left) and time series data of deliveries per client occupation or role (right).

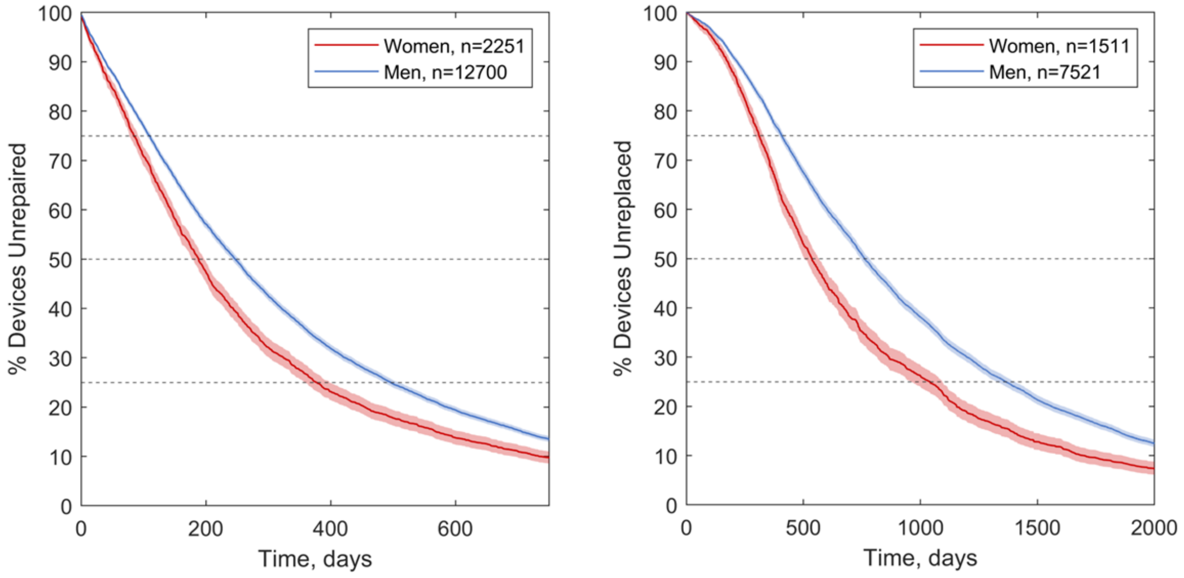

Figure S5: Kaplan-Meier estimates of time to repair (left) and replacement (right) of prosthetic devices, for active clients who were women and men. Shaded zones indicate 95% C.I. and dashed lines enable the 25th percentile, median and 75th percentile times to repair or replacement to be compared between groups.

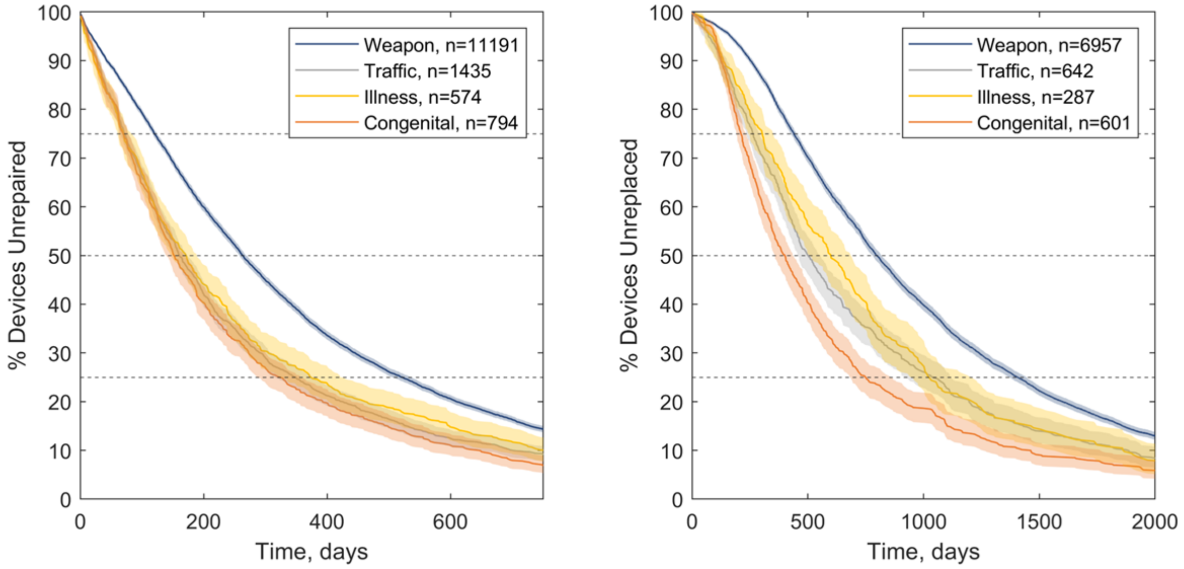

Figure S6: Kaplan-Meier estimates of time to repair (left) and replacement (right) of prosthetic devices, by reason for limb absence in active clients. Shaded zones indicate 95% C.I. and dashed lines enable the 25th percentile, median and 75th percentile times to repair or replacement to be compared between groups.

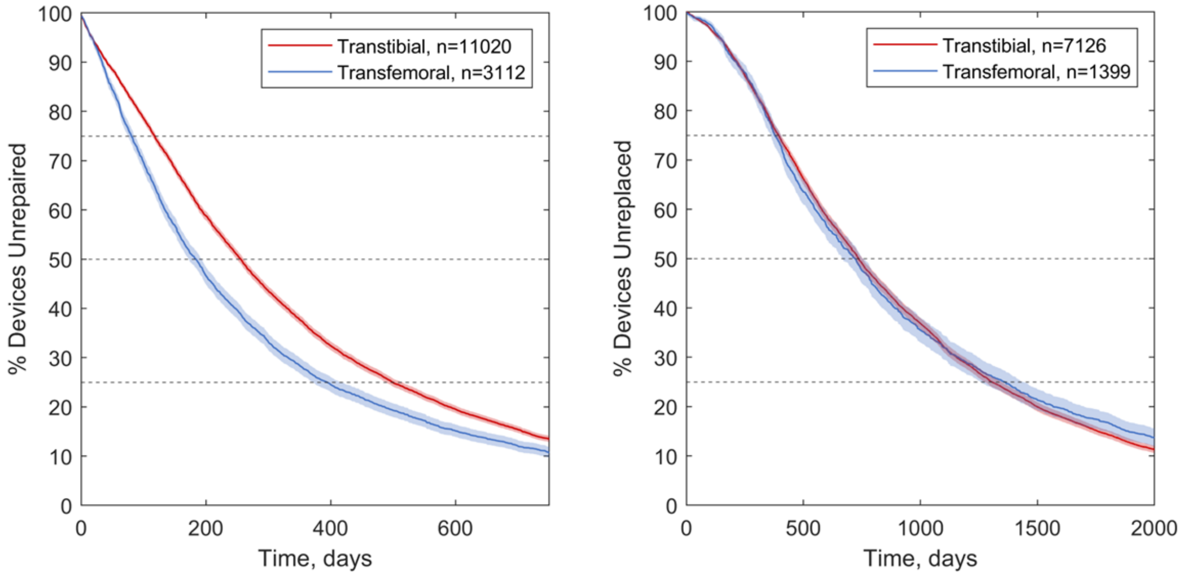

Figure S7: Kaplan-Meier estimates of time to repair (left) and replacement (right) of prosthetic devices, for device type in active clients. Shaded zones indicate 95% C.I. and dashed lines enable the 25th percentile, median and 75th percentile times to repair or replacement to be compared between groups.

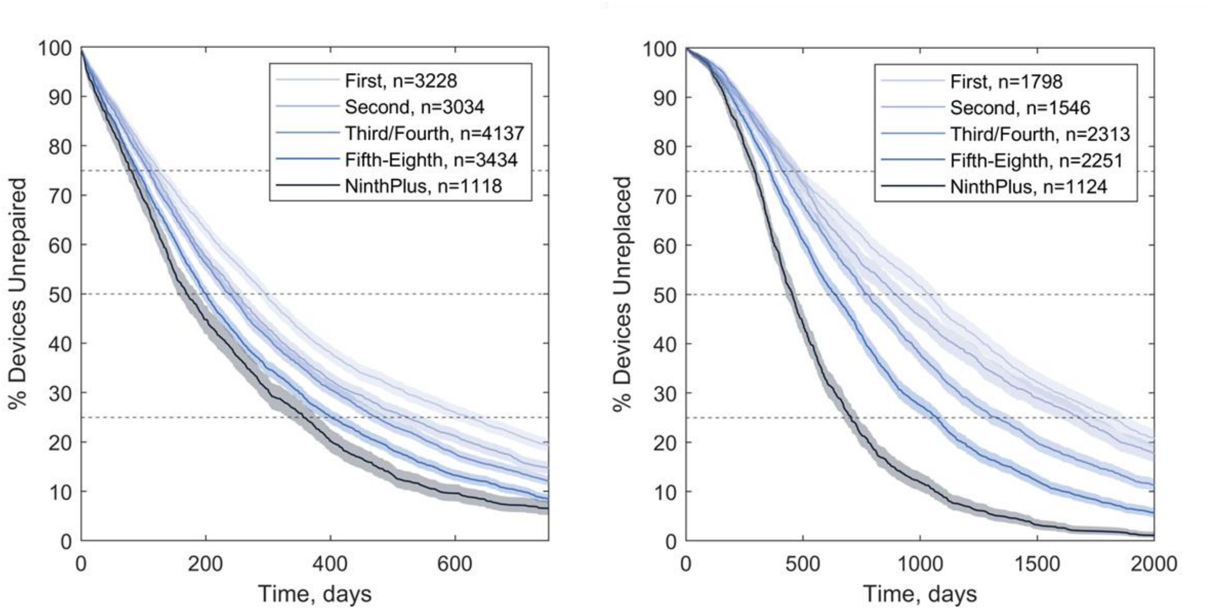

Figure S8: Kaplan-Meier estimates of time to repair (left) and replacement (right) of prosthetic devices, for devices categorised by the client's device delivery number. Shaded zones indicate 95% C.I. and dashed lines enable the 25th percentile, median and 75th percentile times to repair or replacement to be compared between groups.

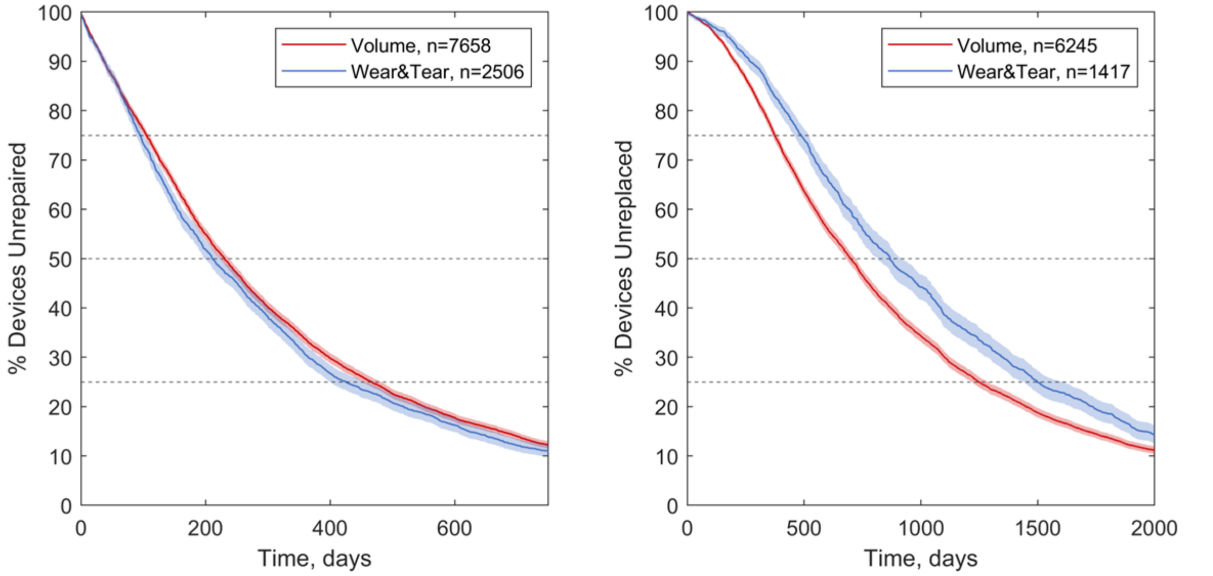

Figure S9: Kaplan-Meier estimates of time to repair (left) and replacement (right) of prosthetic devices, for the two most frequent reasons for replacement. Shaded zones indicate 95% C.I. and dashed lines enable the 25th percentile, median and 75th percentile times to repair or replacement to be compared between groups.

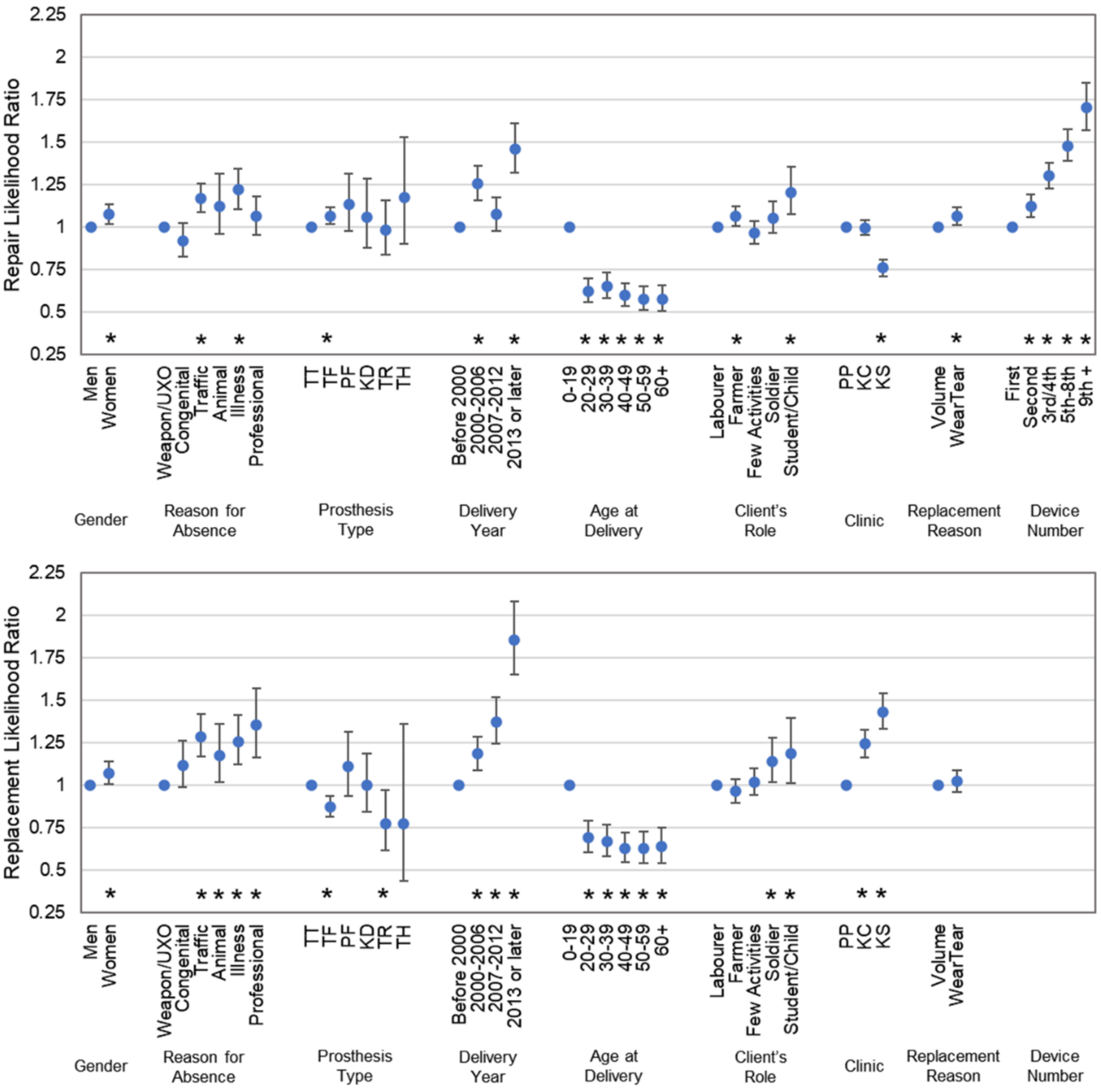

*Figure S10: The likelihood ratio of prosthetic device repairs (top) and replacements (bottom), for different population groups. Error bars represent 95% Confidence Interval. Within groups, the reference case is chosen as the most frequent (for e.g. gender, reason for limb absence), or earliest (for e.g. decade of birth or number of device). * denotes statistical significance at p<0.05.*

Appendix 2: Sensitivity analysis for estimating missing device user occupation

Missing device user occupation data estimated from any occupation reported for their preceding device(s). Sensitivity analysis confirmed that this estimation produced no statistically significant change in survivorship for any occupation group, in comparison to excluding the devices with missing user occupations.

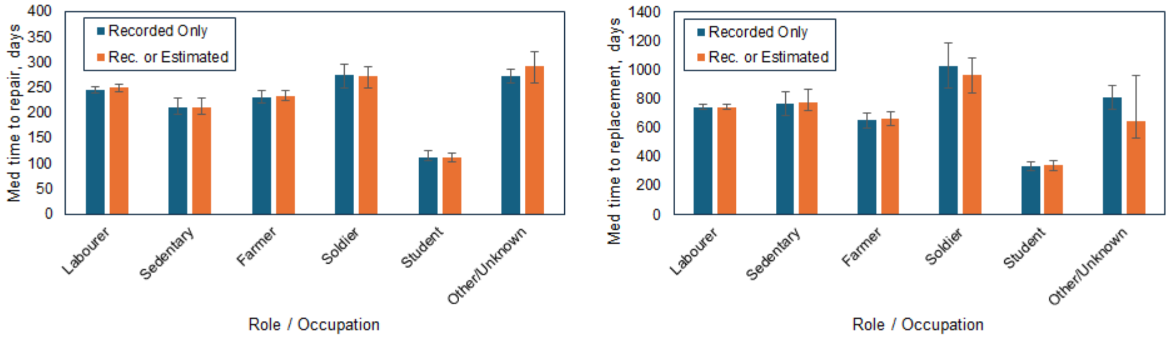

Figure S11: Median and 95% Confidence Interval times to device repair (left) and replacement (right) by either leaving out devices which had missing users' occupations ('Recorded Only') or estimating the device's user's occupation from that recorded for a previous device ('Rec. or Estimated').
